# Supplementary material for: NDRG2 Expression in Breast Cancer Cells Downregulates PD-L1 Expression and Restores T Cell Proliferation in Tumor-Coculture
Source: Cancers (Basel). 2021 Dec 4;13(23):6112. doi: 10.3390/cancers13236112 (PMC8656534; doi:10.3390/cancers13236112)
Supplement: Supplementary file 1 [file cancers-13-06112-s001.zip › cancers-1497161-supplementary.pdf]

cancers MDPI

Supplementay Material: NDRG2 Expression in Breast Cancer Cells Downregulates PD-L1 Expression and Restores T Cell Proliferation in Tumor-Coculture

Aram Lee, Soyoung Lim, Juyeong Oh, Jihyun Lim, Young Yang, Myeong-Sok Lee and Jong-Seok Lim

Table S1. Human RT-PCR primer sequences.

Gene	Sequence '5 → 3'
PD-1 (sense)	AAGGCGCAGATCAAAGAGAG
PD-1 (anti-sense)	TCCGCTAGGAAAGACAATGG
PD-L1 (sense)	AAACAATTAGACCTGGCTG
PD-L1 (anti-sense)	CAAGTCCTGAGTGGTAAGA
PD-L2 (sense)	GGGACGAAGGACAGTACCAA
PD-L2 (anti-sense)	TGCTGGCCAAAGTAAGTTCC
NDRG2 (sense)	GGATTCATGGCGGAGCTGCAGGAGG
NDRG2 (anti-sense)	GAATTCTCAACAGGAGACCTCCATGGT
β-actin (sense)	CCACACCTTCTACAATGAGC
β-actin (anti-sense)	TGAGGTAGTCAGTCAGGTCC

Table S2. Human quantitative PCR primer sequences.

Gene	Sequence '5 → 3'
NDRG2 (sense)	TCTGTCACTTTCACTGTCTA
NDRG2 (anti-sense)	CCAGAGATGGGTACTGATAT
PD-L1 (sense)	GGTGCCGACTACAAGCGAAT
PD-L1 (anti-sense)	AGCCCTCAGCCTGACATGTC
PD-1 (sense)	AGCCCTCAGCCTGACATGTC
PD-1 (anti-sense)	TTTCAGGAATGGGTTCCAAG
GAPDH (sense)	CTGGGCTACACTGAGCACCAG
GAPDH (anti-sense)	CCAGCGTCAAAGGTGGAG

The uncropped blots and molecular weight markers

Figure 1E

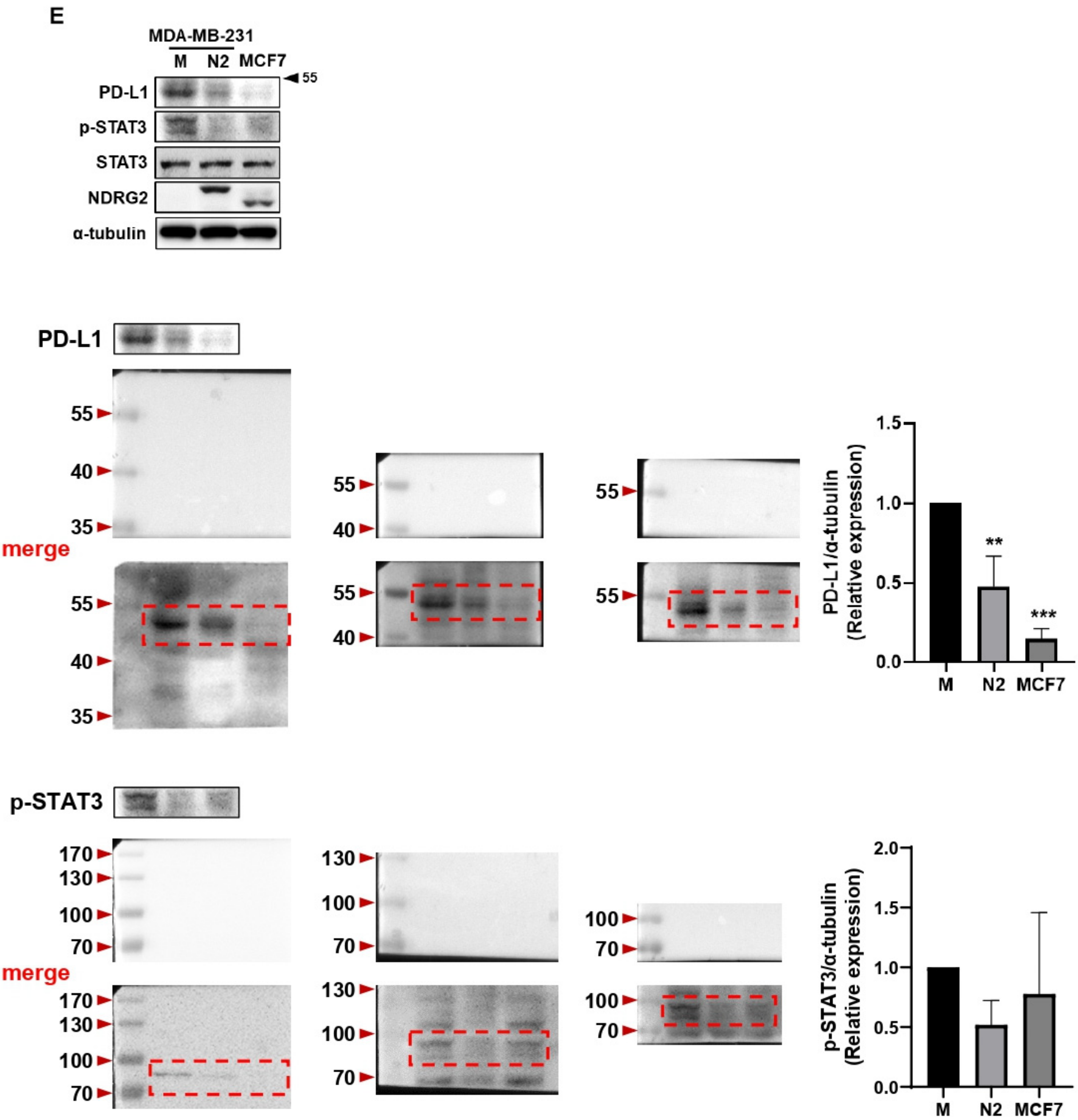

Figure 1E

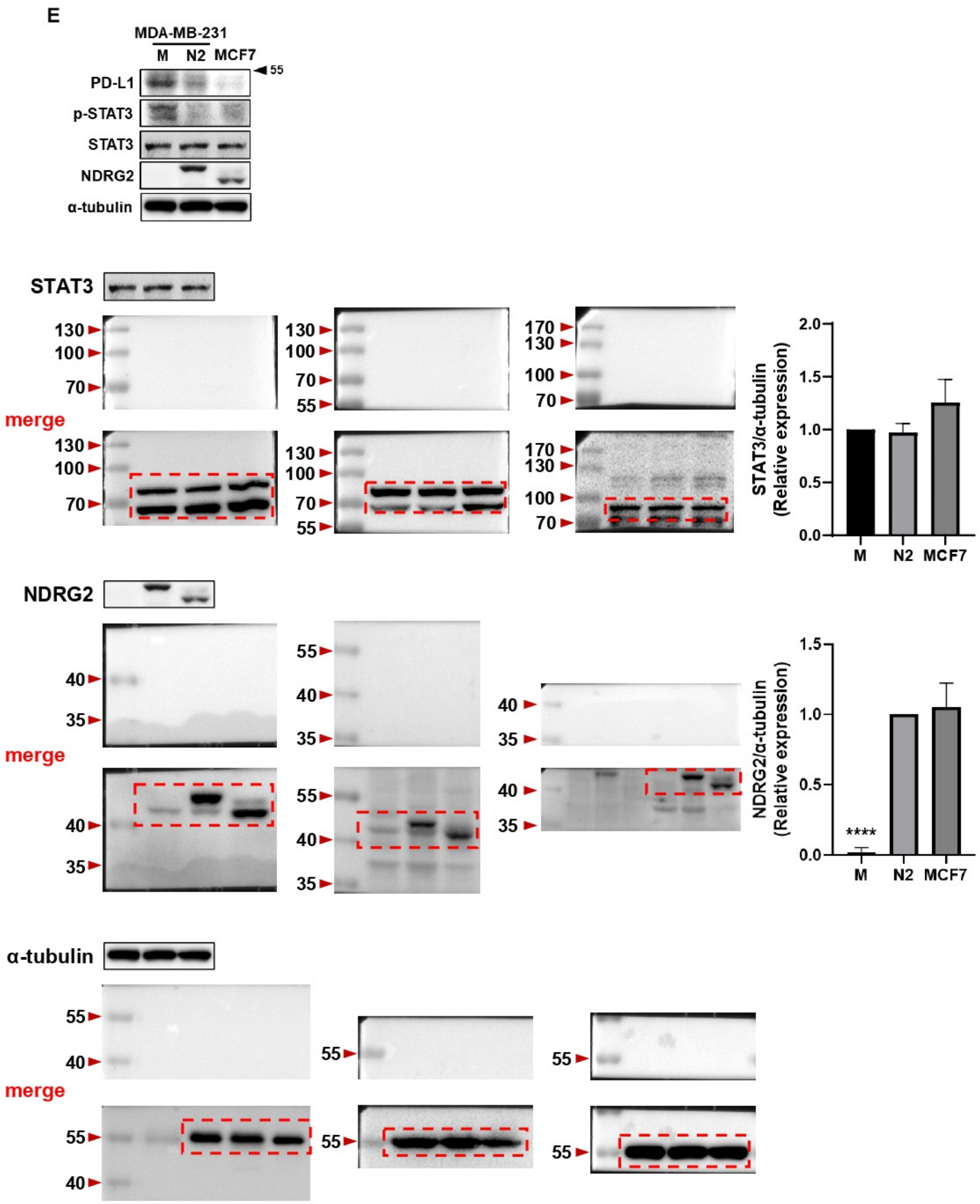
E
MDA-MB-231
M
N2
MCF7
55
PD-L1
p-STAT3
STAT3
NDRG2
α-tubulin
STAT3
merge
130
100
70
55
170
STAT3/α-tubulin
(Relative expression)
2.0
1.5
1.0
0.5
0.0
NDRG2
40
35
NDRG2/α-tubulin
****
α-tubulin

Figure 2A

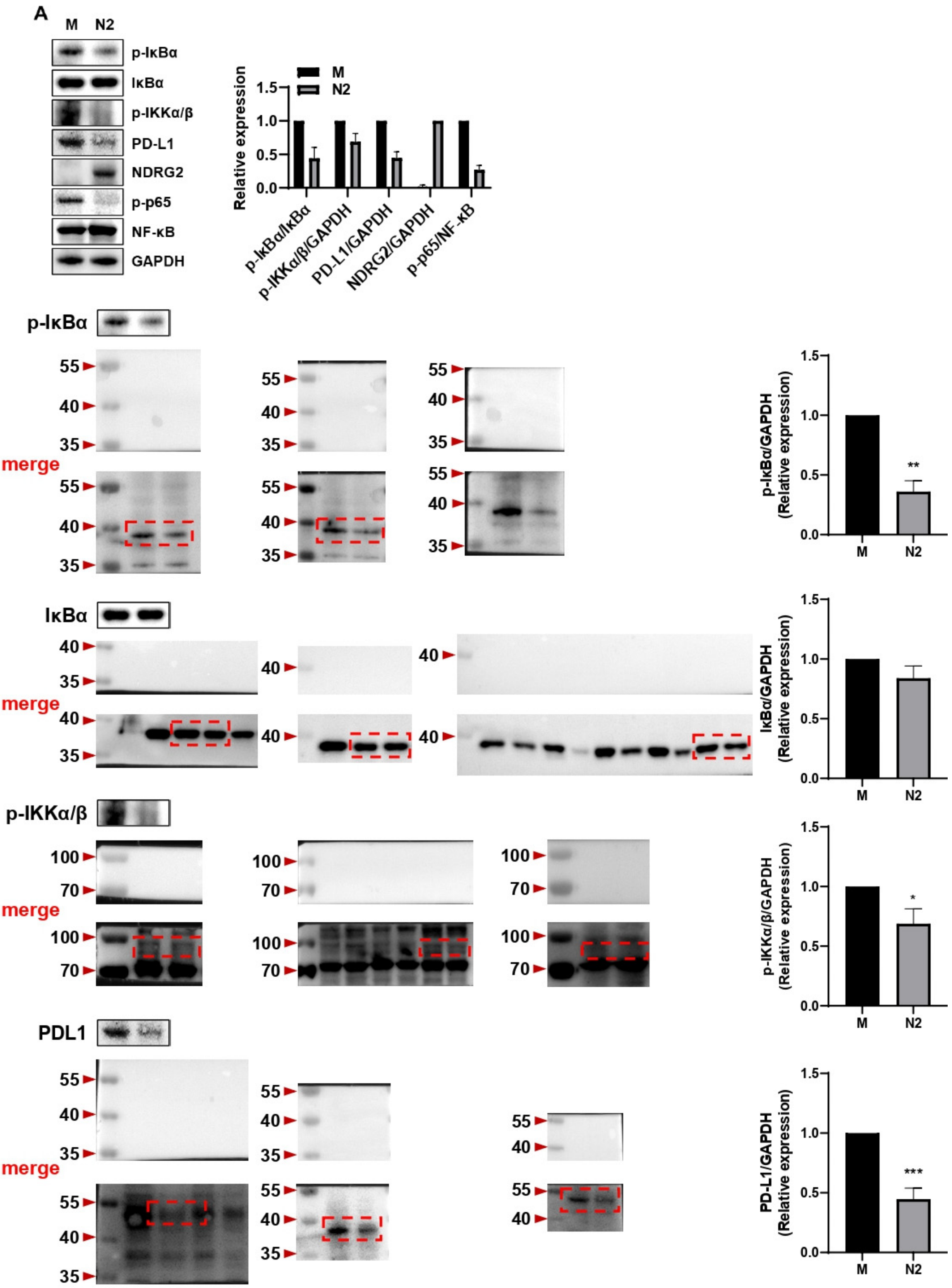

A
M
N2
p-IκBα
IκBα
p-IKKα/β
PD-L1
NDRG2
p-p65
NF-κB
GAPDH
Relative expression
p-IκBα/IκBα
p-IKKα/β/GAPDH
PD-L1/GAPDH
NDRG2/GAPDH
p-p65/NF-κB
merge
PDL1
p-IκBα/GAPDH (Relative expression)
IκBα/GAPDH (Relative expression)
p-IKKα/β/GAPDH (Relative expression)
PD-L1/GAPDH (Relative expression)
**
*
***

Figure 2A

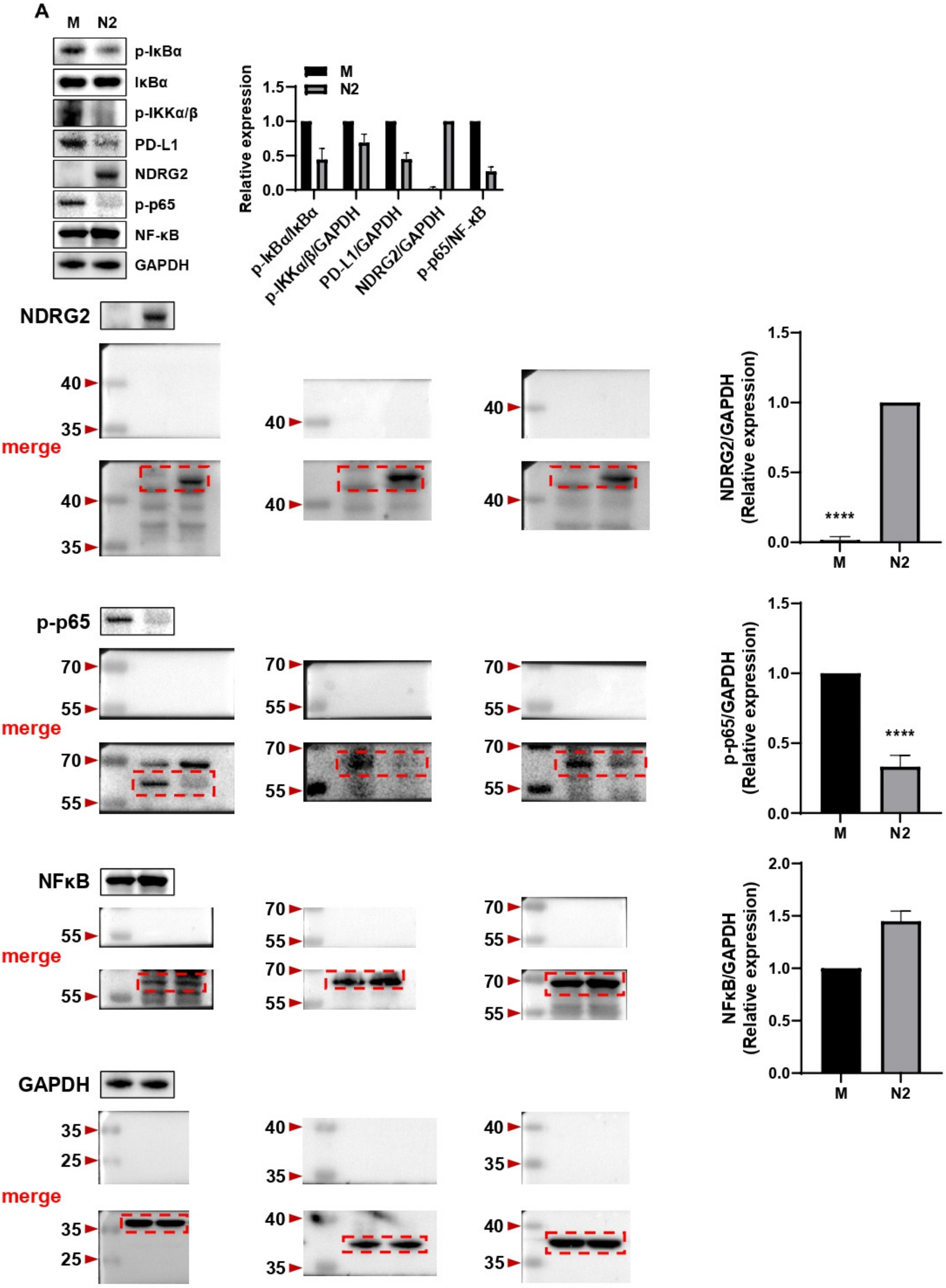

A
M
N2
p-IκBα
IκBα
p-IKKα/β
PD-L1
NDRG2
p-p65
NF-κB
GAPDH
Relative expression
p-IκBα/IκBα
p-IKKα/β/GAPDH
PD-L1/GAPDH
NDRG2/GAPDH
p-p65/NF-κB
NDRG2
merge
NDRG2/GAPDH (Relative expression)
p-p65
p-p65/GAPDH (Relative expression)
NFκB
NFκB/GAPDH (Relative expression)
GAPDH

Figure 2B

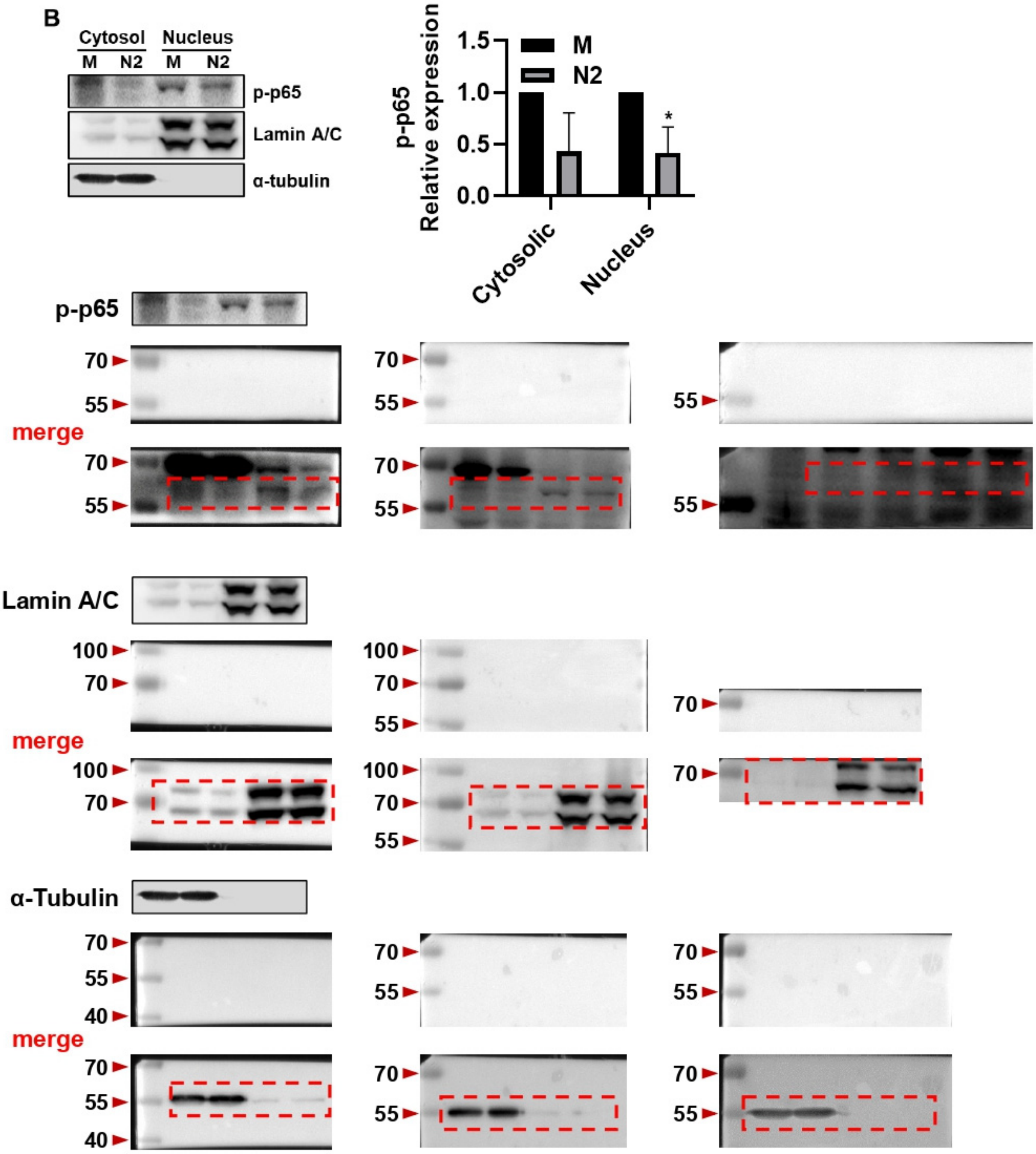
B
Cytosol
Nucleus
M
N2
M
N2
p-p65
Lamin A/C
α-tubulin
p-p65
Relative expression
1.5
1.0
0.5
0.0
M
N2
*
Cytosolic
Nucleus
p-p65
70
55
merge
Lamin A/C
100
α-Tubulin
40

Figure 2C

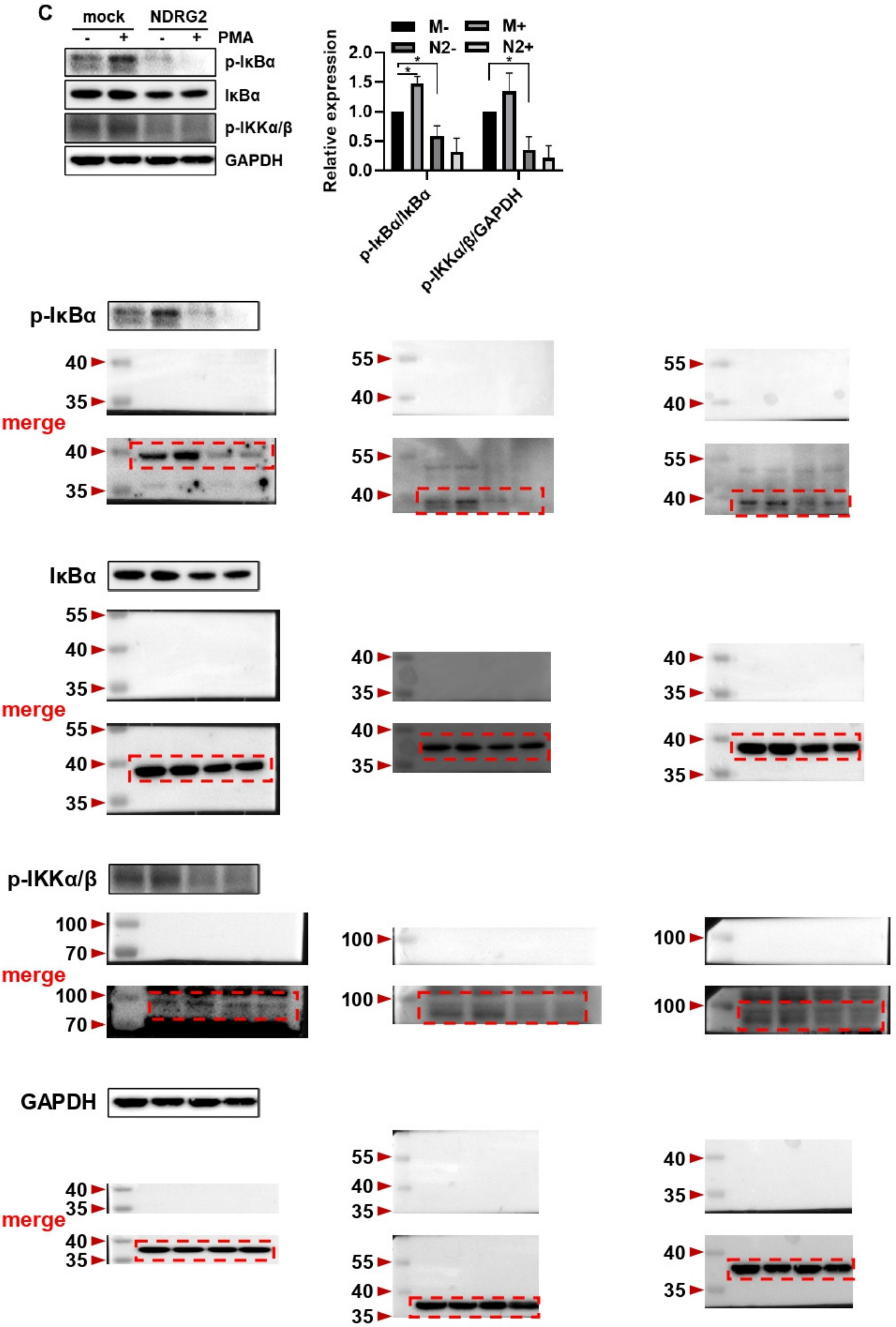

C
mock
NDRG2
PMA
p-IκBα
IκBα
p-IKKα/β
GAPDH
M-
M+
N2-
N2+
Relative expression
2.0
1.5
1.0
0.5
0.0
p-IκBα/IκBα
p-IKKα/β/GAPDH
merge
100
70
55
40
35

Figure 2D

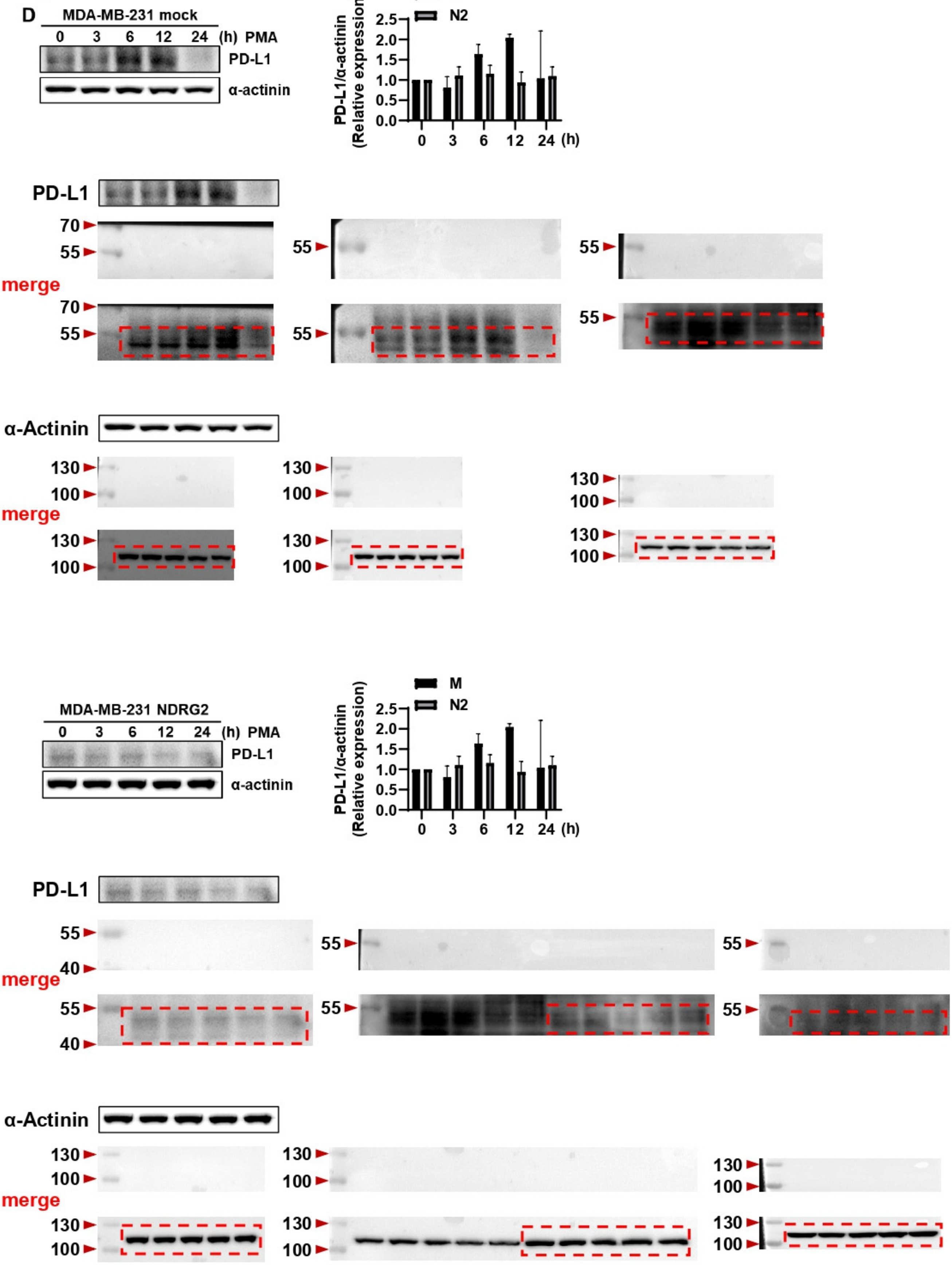
D
MDA-MB-231 mock
0 3 6 12 24 (h) PMA
PD-L1
α-actinin
M
N2
PD-L1/α-actinin (Relative expression)
2.5
2.0
1.5
1.0
0.5
0.0
0 3 6 12 24 (h)
PD-L1
70
55
merge
α-Actinin
130
100
MDA-MB-231 NDRG2
0 3 6 12 24 (h) PMA
PD-L1
α-actinin
40

Figure 3B

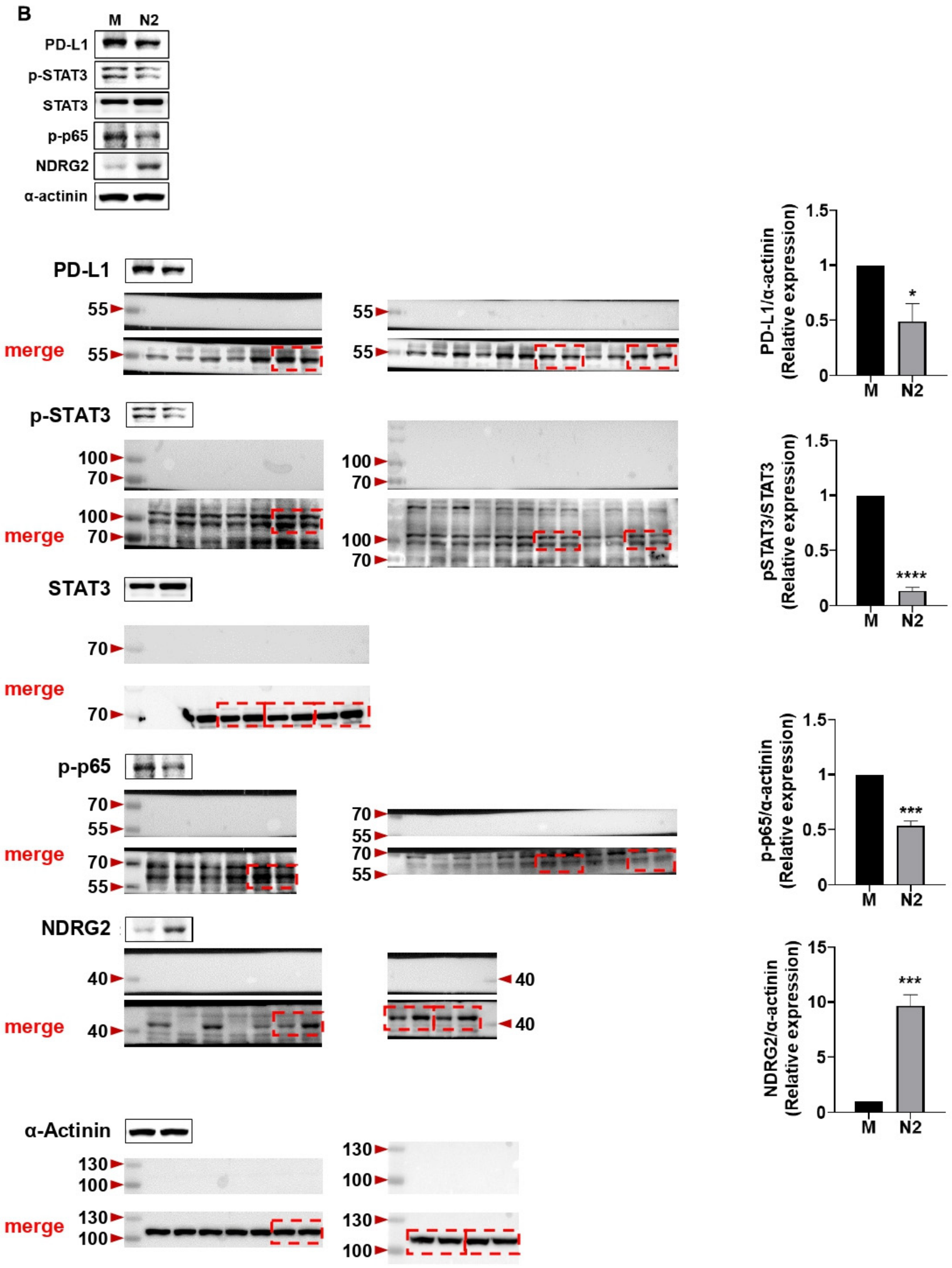
B
M
N2
PD-L1
p-STAT3
STAT3
p-p65
NDRG2
α-actinin
PD-L1
55
merge
55
p-STAT3
100
70
merge
STAT3
70
p-p65
NDRG2
40
α-Actinin
130
PD-L1/α-actinin
(Relative expression)
pSTAT3/STAT3
(Relative expression)
p-p65/α-actinin
(Relative expression)
NDRG2/α-actinin
(Relative expression)
1.5
1
0.5
0
15
10
5
*
****
***

Figure 5A

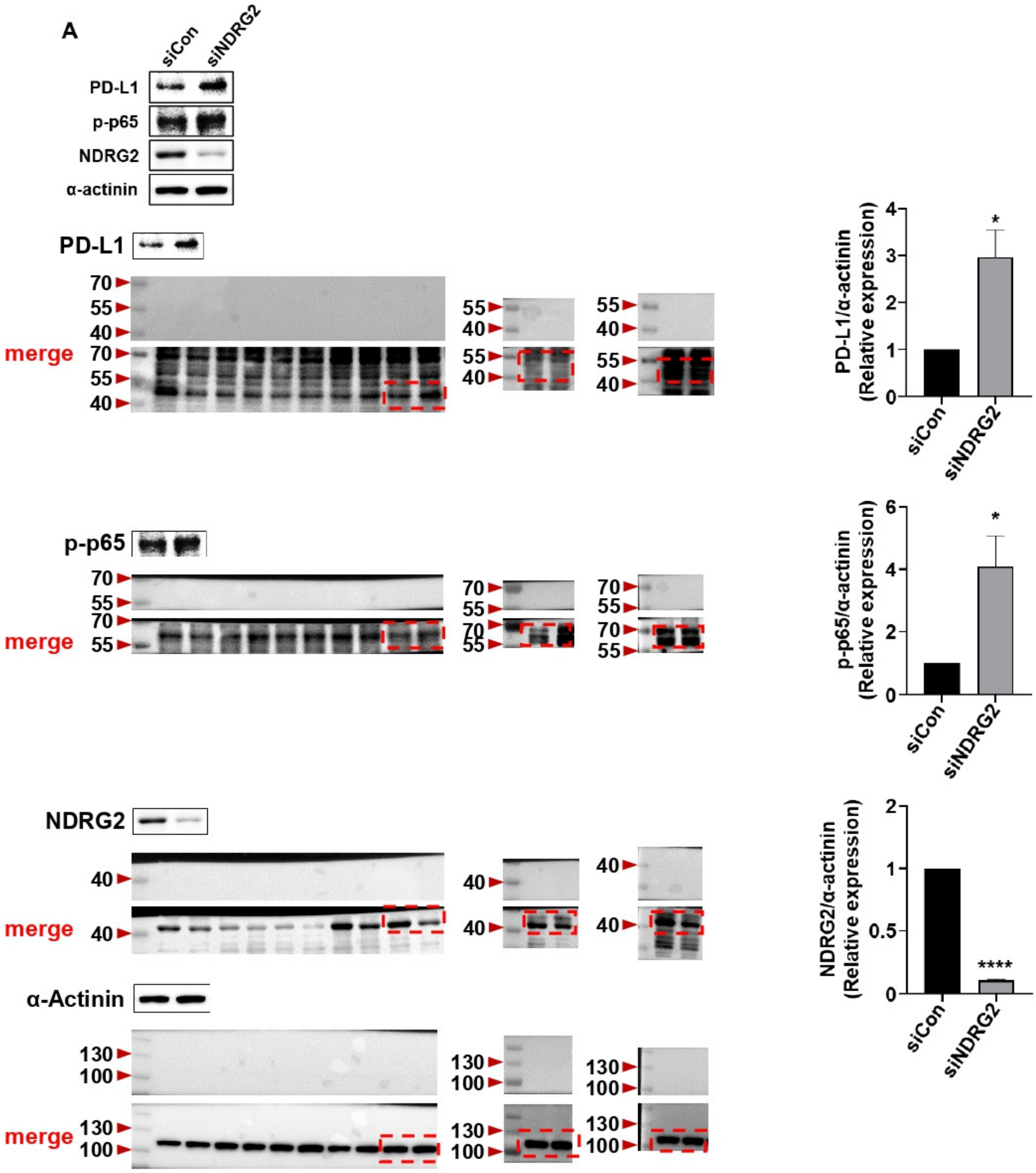

A
siCon
siNDRG2
PD-L1
p-p65
NDRG2
α-actinin
merge
70
55
40
130
100
α-Actinin
PD-L1/α-actinin
(Relative expression)
p-p65/α-actinin
(Relative expression)
NDRG2/α-actinin
(Relative expression)
0
1
2
3
4
6
0.5
*
****
